# Association of ACEi/ARB Use and Clinical Outcomes of COVID-19 Patients With Hypertension

**DOI:** 10.3389/fcvm.2021.577398

**Published:** 2021-05-31

**Authors:** Jing Ma, Xiaowei Shi, Jiong Yu, Feifei Lv, Jian Wu, Xinyu Sheng, Qiaoling Pan, Jinfeng Yang, Hongcui Cao, Lanjuan Li

**Affiliations:** ^1^State Key Laboratory for the Diagnosis and Treatment of Infectious Diseases, National Clinical Research Center for Infectious Diseases, The First Affiliated Hospital, Zhejiang University School of Medicine, Hangzhou, China; ^2^Collaborative Innovation Center for Diagnosis and Treatment of Infectious Diseases, Hangzhou, China; ^3^Department of Laboratory Medicine, The First Affiliated Hospital, Zhejiang University School of Medicine, Hangzhou, China; ^4^Department of Laboratory Medicine, The First People's Hospital of Yancheng City, Yancheng, China

**Keywords:** angiotensin-converting enzyme inhibitors, angiotensin receptor blockers, COVID-19, hypertension, SARS-CoV-2

## Abstract

**Objectives:** Evidence has shown that angiotensin-converting enzyme 2 (ACE2), which can be upregulated after angiotensin-converting enzyme inhibitor (ACEi) and angiotensin receptor blocker (ARB) treatment, may play a dual role in the pathogenesis and progression of coronavirus disease 2019 (COVID-19). We aimed to assess the association between the use of ACEi/ARB and the outcome of COVID-19 patients with preexisting hypertension in non-endemic areas.

**Methods:** From January 17, 2020, to February 19, 2020, 286 patients with hypertension were enrolled in this retrospective study out of 1,437 COVID-19 patients from 47 centers in Zhejiang and Jiangsu Province. The composite endpoints consisted of mechanical ventilation, intensive care unit (ICU) admission, or death. Cox proportional hazards analysis was performed to assess the association between ACEi/ARB and clinical outcomes of COVID-19 patients with hypertension.

**Results:** In the main analysis, 103 patients receiving ACEi/ARB were compared with 173 patients receiving other regimens. Overall, 44 patients (15.94%) had an endpoint event. The risk probability of crude endpoints in the ACEi/ARB group (12.62%) was lower than that in the non-ACEi/ARB group (17.92%). After adjusting for confounding factors by inverse probability weighting, the results showed that the use of ACEi/ARB reduced the occurrence of end events by 47% [hazard ratio (HR) = 0.53; 95% CI, 0.34–0.83]. Similar results were obtained in multiple sensitivity analyses.

**Conclusions:** In this retrospective study, among COVID-19 patients with hypertension, the use of ACEi/ARB is not associated with an increased risk of disease severity compared with patients without ACEi/ARB. The trends of beneficial effects of ACEi/ARB need to be further evaluated in randomized clinical trials.

## Introduction

The novel coronavirus disease 2019 (COVID-19) is spreading worldwide, with an increasing number of confirmed cases and deaths, and has received widespread attention from the World Health Organization. It is currently known that COVID-19 patients with hypertension are prone to have poor clinical outcomes (1). Angiotensin-converting enzyme inhibitors (ACEis) and angiotensin receptor blockers (ARBs) are widely used in the treatment of hypertension. In animal studies, the expression of angiotensin-converting enzyme 2 (ACE2) is upregulated after ACEi and ARB treatment (2). Intriguingly, ACE2 plays a dual role in COVID-19 progression. On one hand, severe acute respiratory syndrome coronavirus 2 (SARS-CoV-2) binds with ACE2 to enter the host cell during invasion (3), resulting in a decrease in ACE2 and subsequently causing vasoconstriction. Based on this, patients with a medical history of ACEi/ARB may be more likely to suffer from SARS-COV-2 infection and severe progression due to elevated ACE2 expression, and it has proposed that alternative treatments be sought for those with a high risk of infection (4). On the other hand, evidence from various acute respiratory distress syndrome (ARDS) animal models showed that exogenous ACE2 supplementation can reduce inflammation and increase oxygenation (2). The absence of the protective role of ACE2 may lead to renin–angiotensin system (RAS) dysregulation and potentially give rise to extensive endothelial dysfunction and acute lung injury (5). Thus, ACEi/ARB may, in turn, be beneficial as it prevents RAS overactivation by increasing ACE2 expression, reducing the risk of acute lung injury and acute respiratory distress syndrome.

Several studies have indicated that ACEi/ARB use was associated with decreased mortality in patients with COVID-19 (6–8), but most studies supported that ACEi/ARB use was not related to disease severity (1, 8–12). A recent meta-review of ours also concluded that ACEi/ARB therapy was associated with a lower risk of mortality compared to those who have non-ACEi/ARB antihypertensive drugs but not associated with a higher risk of COVID-19 severity (13). Indeed, the use of ACEi/ARB in patients with COVID-19 remains controversial. And very few large-sample studies are conducted outside the pandemic area in China (14, 15). Therefore, the present study aimed to assess the association between ACEi/ARB use and its impact on the risk of severity in COVID-19 patients with hypertension in non-endemic areas by inverse probability of treatment weighting (IPTW) analysis.

## Methods

### Patients

Patients diagnosed with COVID-19 were recruited for this multicenter retrospective study from 47 centers in Zhejiang and Jiangsu Province between January 17, 2020, and February 19, 2020. All patients enrolled in this study were diagnosed with hypertension and COVID-19 according to the diagnostic criteria of the National Health Commission. This study was approved by the Ethics Committee of the First Affiliated Hospital, College of Medicine, Zhejiang University (No. IIT20200005C), and complied with the ethical guidelines of the Declaration of Helsinki. Written informed consent was waived, as this study was conducted on an emerging infectious disease and the researchers analyzed only anonymous data.

### Data Collection

Epidemiological, demographic, comorbidities, clinical, laboratory, time from illness onset to hospital admission, time to the first dose of antiviral delivery, chest radiological findings at admission, and outcome data were collected from patients' electronic medical records, with verification by independent doctors. The COVID-19 cases were all confirmed by throat swab specimens from the upper respiratory tract using sequencing or RT-PCR assay. Clinical outcomes were followed up to March 15, 2020.

### Definition

The patients were classified into four types: mild, moderate, severe, and critical type according to the guidelines on the Diagnosis and Treatment of COVID-19 by the National Health Commission (16). All patients taking ACEi and ARB antihypertensive drugs, whether combined or not, were classified in the ACEi/ARB group based on their main complaint at admission. In principle, the antihypertensive regimens remained the same as the drugs used by patients before admission. Hypertension grades were defined as Grade 1, Grade 2, and Grade 3 according to 2018 guidelines of the European Society of Hypertension (ESH). The onset of COVID-19 was defined as the time when symptoms were first noticed. The endpoint of this study was defined as a composite measure consisting of mechanical ventilation, intensive care unit (ICU) admission, or death. Briefly, the endpoint represented at least one criterion: respiratory failure occurs and mechanical ventilation is required, develops other organ failures and needs ICU monitoring and treatment, or death (17). If the patient met several criteria for the event, the calculation will be based on the time of the first criterion appearance and follow-up until the patient was discharged.

### Statistical Analysis

Continuous variables were expressed as medians and interquartile range (IQR) 25–75% and were compared by *t-*test or Mann–Whitney U-test. Categorical variables were expressed as percentages and tested with chi-square test or Fisher's exact test. To assess the association between ACEi/ARB use and clinical outcomes of COVID-19 patients, our main analysis compared the 103 participants who received ACEi/ARB with the 173 who received other regimens. Cox proportional hazards regression models were used to assess the association between ACEi/ARB use and the composite endpoint of intubation, ICU admission, or death. The primary analyses adjusted for benchmark covariates, including sex, age, body mass index (BMI), smoking status, duration from onset to admission, C-reactive protein (CRP), treatment of antivirus drugs, clinical type on admission, grade of hypertension, and comorbidities. The main analysis was performed by IPTW to minimize the effect of ACEi/ARB use selection bias and to control for potential confounding factors (18), which included the same covariates as the Cox regression model (19). The estimated propensity score was obtained as the predicted probability of each subject treated with ACEi/ARB. The standardized differences were examined to assess the covariates included in estimating propensity scores before and after weighting, with a statistic <10% indicating a clinically meaningful balance between the two groups (19). Missing data were performed through multiple imputations by chained equations using the other variables available (20). All statistical analyses were performed by Statistical Package for the Social Sciences version 19.0 (International Business Machines Corporation, Armonk, NY) and R version 3.4 (R Foundation, Vienna, Austria). All tests were two–tailed, and *p* < 0.05 was considered to indicate statistical significance.

### Other Sensitivity Analyses

In addition, we conducted eight prespecified subgroups and sensitivity analyses to evaluate the robustness of the composite endpoint: (1) age (age <60 vs. ≥60 years), (2) sex (male vs. female), (3) median value of onset to admission (<4 vs. ≥4 days), (4) CRP (<8 vs. ≥8 mg/L), (5) BMI (<25 vs. ≥25 kg/m^2^), (6) presence of diabetes (yes vs. no), (7) clinical type on admission (mild/moderate vs. severe), (8) grade of hypertension (1 vs. 2 vs. 3).

Second, all patients eligible for the study were analyzed, and those without any antihypertensive drugs were analyzed in the control group.

## Results

### Clinical Characteristics and Symptoms on Admission

**Figure 1:**
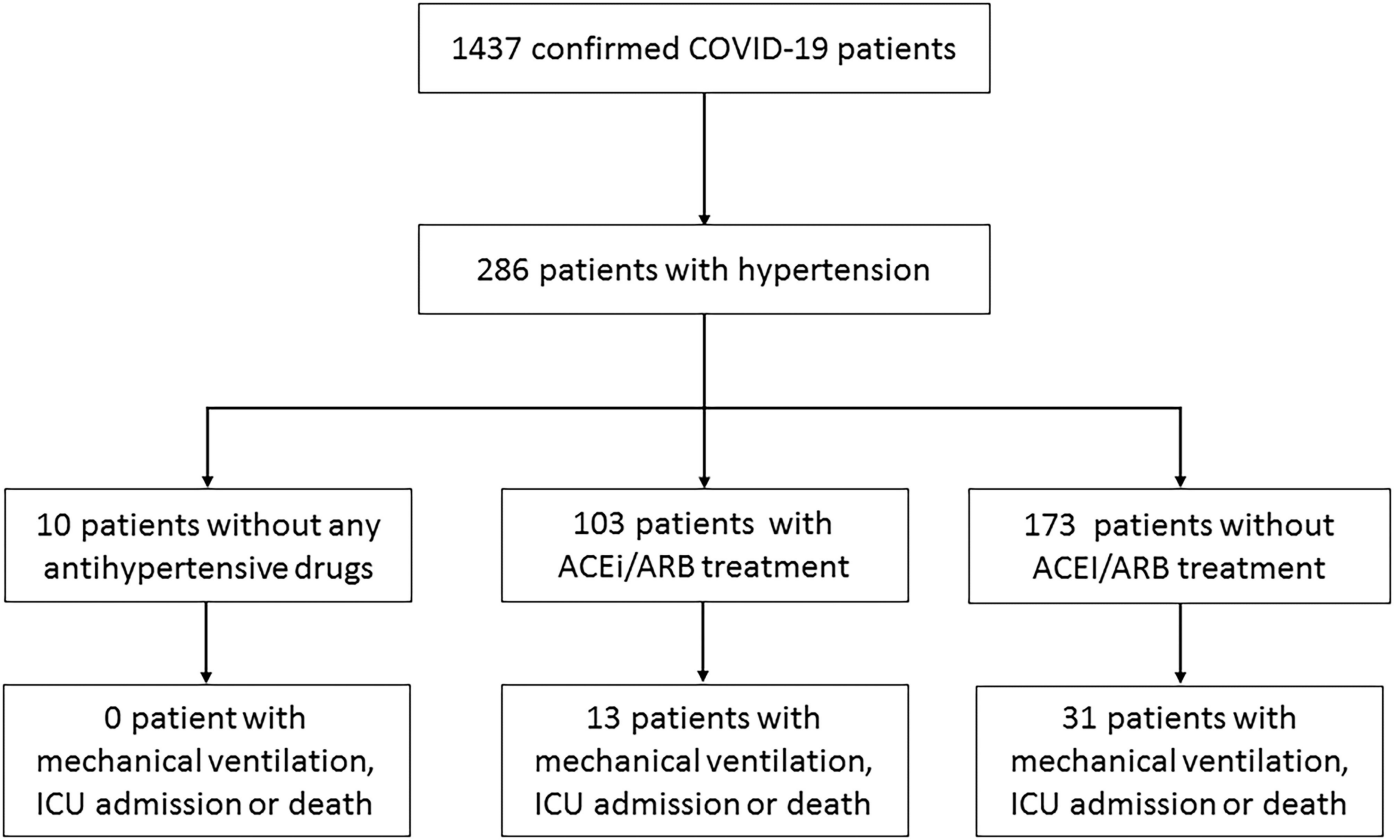
Flowchart of patient selection.

From January 17, 2020, to February 19, 2020, 286 patients with hypertension were enrolled in this study out of 1,437 COVID-19 patients in 47 centers of Zhejiang and Jiangsu Province (Figure 1). Among the patients, 103 patients received ACEi/ARB therapy, including 12 with ACEi, 91 with ARB, and 46 combined with other types of drugs. Besides, 173 patients were treated with other regimens, including 143 (82.66%) with calcium channel blockers, 20 (11.56%) with beta-blockers, 40 (22.73%) with diuretics, and three (1.73%) with centrally acting agents (**Table 2**) and 10 without any antihypertensive drugs.

Clinical characteristics of patients from the ACEi/ARB group and other regimens group are shown in Table 1. There were no significant differences in either age or sex between the two groups (*p* > 0.05). Fever and cough were the main symptoms in the ACEi/ARB group and other regimens group, and the proportion in the two groups had no significant differences. In addition to hypertension, 97 (35.14%) patients had at least one comorbidity other than hypertension. The ACEi/ARB group included 22 cases of diabetes, five cases of cardiovascular diseases, and nine cases of chronic liver disease. And there were 32 with diabetes, 21 with cardiovascular disease, and 13 with chronic liver disease in the non-ACEi/ARB group. There are significant differences in the grade of hypertension: the proportion of grade 1 hypertension was 54 (52.43%) in the ACEi/ARB group vs. 109 (63.01%) in the non-ACEi/ARB group; grade 2, 38 (36.89%) vs. 33 (19.08%); and grade 3, 11 (10.68%) vs. 31 (17.92%), respectively (*p* = 0.003) (Table 1). The results of the remaining laboratory tests were shown in Supplementary Table 1.

**Table 1:** Characteristics of COVID-19 patients with hypertension with or without ACEi/ARB therapy.

**Characteristic**	**Non-ACEi/ARB**	**ACEi/ARB**	***p-*value**
	**(*n =* 173)**	**(*n =* 103)**	
Age (years)	62 (52–68)	59 (52–67)	0.450
BMI (kg/m^2^)^*^	25.53 (23.52–27.28)	24.84 (22.58–27.30)	0.190
Duration from onset to admission (days)	4 (2–7)	5 (3–7)	0.928
Temperatures (°C)	38.00 (37.50–38.50)	38.00 (37.40–38.50)	0.211
Female (%)	76 (43.93%)	55 (53.40%)	0.128
Current smoker (%)	15 (8.67%)	9 (8.74%)	1
**Exposure history**			
Contact with patients (%)	91 (52.60%)	54 (52.43%)	0.978
Cluster (%)	31 (17.92%)	19 (18.45%)	0.912
From Wuhan (%)	50 (28.90%)	34 (33.01%)	0.473
**Symptoms**			
Fever (%)	139 (80.35%)	79 (76.70%)	0.472
Cough (%)	107 (61.85%)	69 (66.99%)	0.390
Expectoration (%)	52 (30.06%)	25 (24.27%)	0.300
Sore throat (%)	15 (8.67%)	13 (12.62%)	0.293
Muscle ache (%)	19 (10.98%)	10 (9.71%)	0.739
Fatigue (%)	43 (24.86%)	26 (25.24%)	0.943
Shortness of breath (%)	19 (10.98%)	12 (11.65%)	0.865
Diarrhea (%)	15 (8.67%)	6 (5.83%)	0.485
Sick (%)	6 (3.47%)	3 (2.91%)	1
Headache (%)	6 (3.47%)	4 (3.88%)	1
**Coexisting comorbidity**			
Cardiovascular diseases (%)	21 (12.14%)	5 (4.85%)	0.055
Diabetes (%)	32 (18.50%)	22 (21.36%)	0.562
COPD (%)	2 (1.16%)	0 (0.00%)	0.530
Asthma (%)	2 (1.16%)	0 (0.00%)	0.530
Cancer (%)	6 (3.47%)	1 (0.97%)	0.263
Chronic liver disease (%)	13 (7.51%)	9 (8.74%)	0.819
Chronic renal disease (%)	3 (1.73%)	3 (2.91%)	0.674
Chest x-ray/CT findings			0.520
Normal (%)	12 (6.94%)	3 (2.97%)	
Unilateral pneumonia (%)	20 (11.56%)	10 (9.90%)	
Bilateral pneumonia (%)	96 (55.49%)	59 (58.42%)	
Multiple mottling and ground-glass opacity (%)	45 (26.01%)	29 (28.71%)	
**Grade of hypertension**			0.003
Grade 1 (%)	109 (63.01%)	54 (52.43%)	
Grade 2 (%)	33 (19.08%)	38 (36.89%)	
Grade 3 (%)	31 (17.92%)	11 (10.68%)	
Severe/critical type on admission (%)	14 (8.09%)	10 (9.71%)	0.663
C-reactive protein (mg/L)^**^	15.53 (4.54–43.11)	15.80 (5.42–34.16)	0.164

*ACEi, angiotensin-converting enzyme inhibitor; ARB, angiotensin receptor blocker; BMI, body mass index; COPD, chronic obstructive pulmonary disease; COVID-19, coronavirus disease 2019*.

* *21 patients with missing data of BMI*.

** *2 patients with missing data of C-reactive protein*.

### The Association of Angiotensin-Converting Enzyme Inhibitor/Angiotensin Receptor Blocker Use With the Composite Endpoints

**Figure 2:**
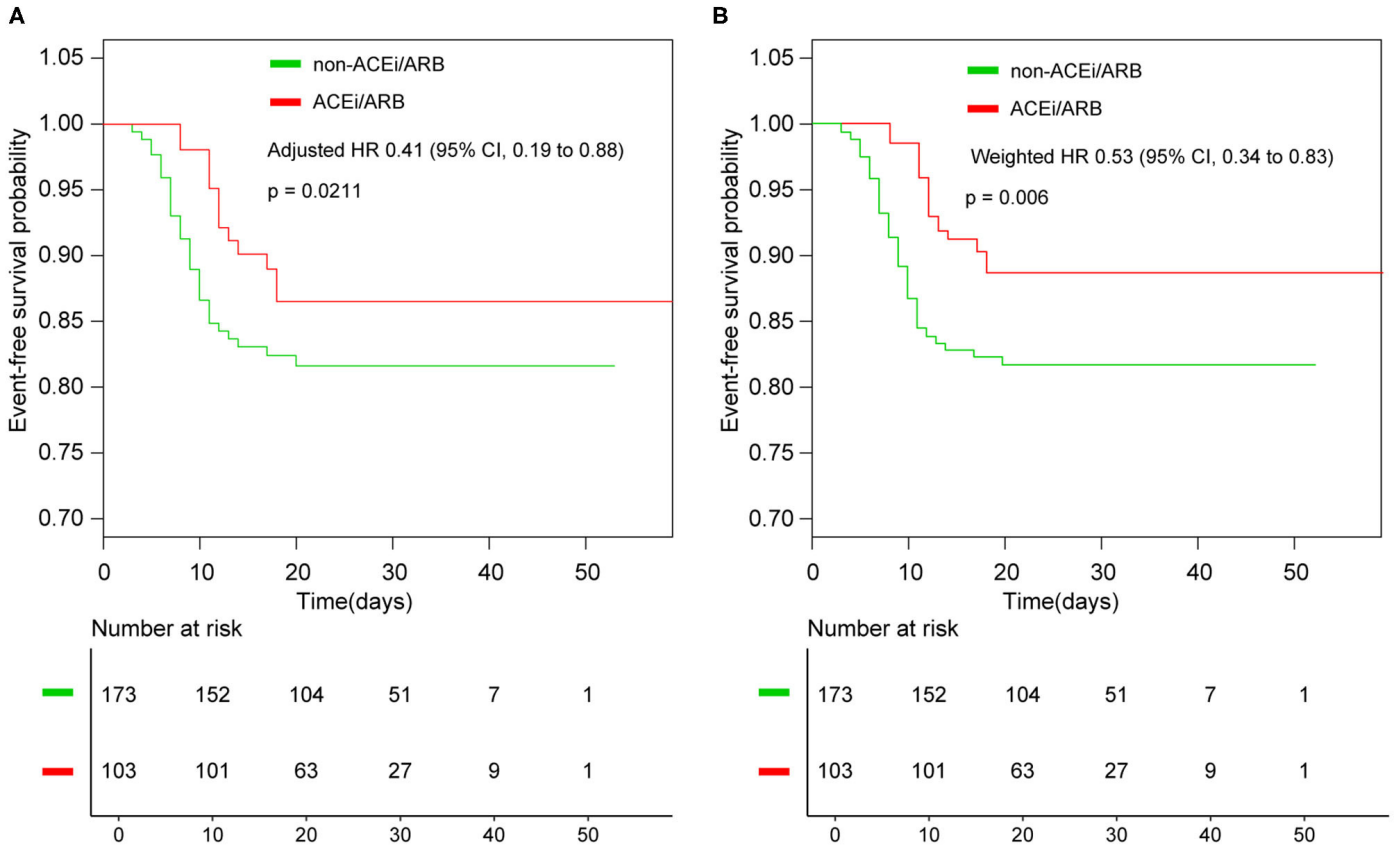
Kaplan–Meier curves for survival without events. **(A)** Kaplan–Meier curves for event-free survival without weighted; **(B)** Kaplan–Meier curves for event-free survival after inverse probability of treatment weighting. ACEi, angiotensin-converting enzyme inhibitor; ARB, angiotensin receptor blocker; HR, hazard ratio.

With a median time of 9 days, 44 patients had disease progression or death in the entire cohort. In detail, two had septic shock and were given vasoactive medications, 34 (12.32%) were admitted to the ICU, 31 (11.23%) received mechanical ventilation, one patient died after intubation, one had lung transplantation, and eight (2.90%) received extracorporeal membrane oxygenation (ECMO) (Table 2). Until March 15, 2020, nine patients had not been discharged, and one of them was in the ACEi/ARB group. The composite endpoints were documented in 13 of 103 (12.62%) patients who received ACEi/ARB therapy compared with 31 of 173 (17.92%) patients in the non-ACEi/ARB group. The rate of events was numerically lower in the ACEi/ARB group than in the non-ACEi/ARB group, but the difference was not significant. The median progression event time was significantly different in the ACEi/ARB group compared with the non-ACEi/ARB group (12 vs. 9 days, *p* = 0.003). In the crude unadjusted analysis, Kaplan–Meier curves for events-free survival showed a hazard ratio (HR) of 0.65 (95% CI, 0.34–1.25; *p* = 0.2002); after adjusting the benchmark covariate, the HR was 0.41 (95% CI, 0.19–0.88; *p* = 0.0211) in the primary multivariable analysis (Figure 2A).

**Table 2:** In-hospital management and outcomes of ACEi/ARB and non-ACEI/ARB groups.

**Variable**	**Non-ACEi/ARB**	**ACEi/ARB**	***p-*value**
	**(*n =* 173)**	**(*n =* 103)**	
Interferon-α	110 (63.58%)	64 (63.37%)	0.971
Oseltamivir	3 (1.73%)	5 (4.95%)	0.149
Fapiravir	6 (3.47%)	5 (5.00%)	0.535
Arbidol	127 (73.41%)	71 (70.30%)	0.579
Lopinavir/ritonavir	96 (55.49%)	61 (60.40%)	0.428
Darunavir	3 (1.84%)	2 (2.27%)	1
Chloroquine phosphate	2 (1.23%)	1 (1.14%)	0.95
Glucocorticoids	60 (34.68%)	33 (32.04%)	0.653
IVIGt	47 (27.17%)	25 (24.27%)	0.596
Antibiotics drug	80 (46.24%)	45 (43.69%)	0.68
Antihypertensive agents			
Calcium channel blockers	137 (82.66%)	47 (45.63%)	<0.001
ACEi	0 (0.00%)	12 (11.65%)	<0.001
ARB	0 (0.00%)	91 (88.35%)	<0.001
Beta-blockers	20 (11.56%)	7 (6.80%)	0.198
Diuretics	34 (19.65%)	12 (11.65%)	0.084
Centrally antihypertensive agents	3 (1.73%)	0 (0.00%)	0.296
Shock	1 (0.55%)	1 (0.97%)	1
Admission to ICU	24 (13.87%)	10 (9.71%)	0.309
Mechanical Ventilation	22 (12.72%)	9 (8.74%)	0.311
Venovenous hemofiltration	3 (1.73%)	1 (0.97%)	1
ECMO	6 (3.47%)	2 (1.94%)	0.714
Lung transplantation	1 (0.97%)	0 (0.00%)	1
Composite endpoint	31 (17.92%)	13 (12.62%)	0.245

*ACEi, angiotensin-converting enzyme inhibitor; ARB, angiotensin receptor blocker; ICU, intensive care unit; ECMO, extracorporeal membrane oxygenation; IVIGt, intravenous immunoglobulin treatment*.

In the IPTW analysis, baseline characteristics were balanced in the two groups (Supplementary Figure 1, Supplementary Table 2). Among the 276 patients in the two groups, the events-free survival was 89.48% in the ACEi/ARB group and 81.85% in the non-ACEi/ARB group; the weighted HR was 0.53 (95% CI, 0.34–0.83; *p* = 0.006; Figure 2B).

### Other Sensitivity Analyses

**Figure 3:**
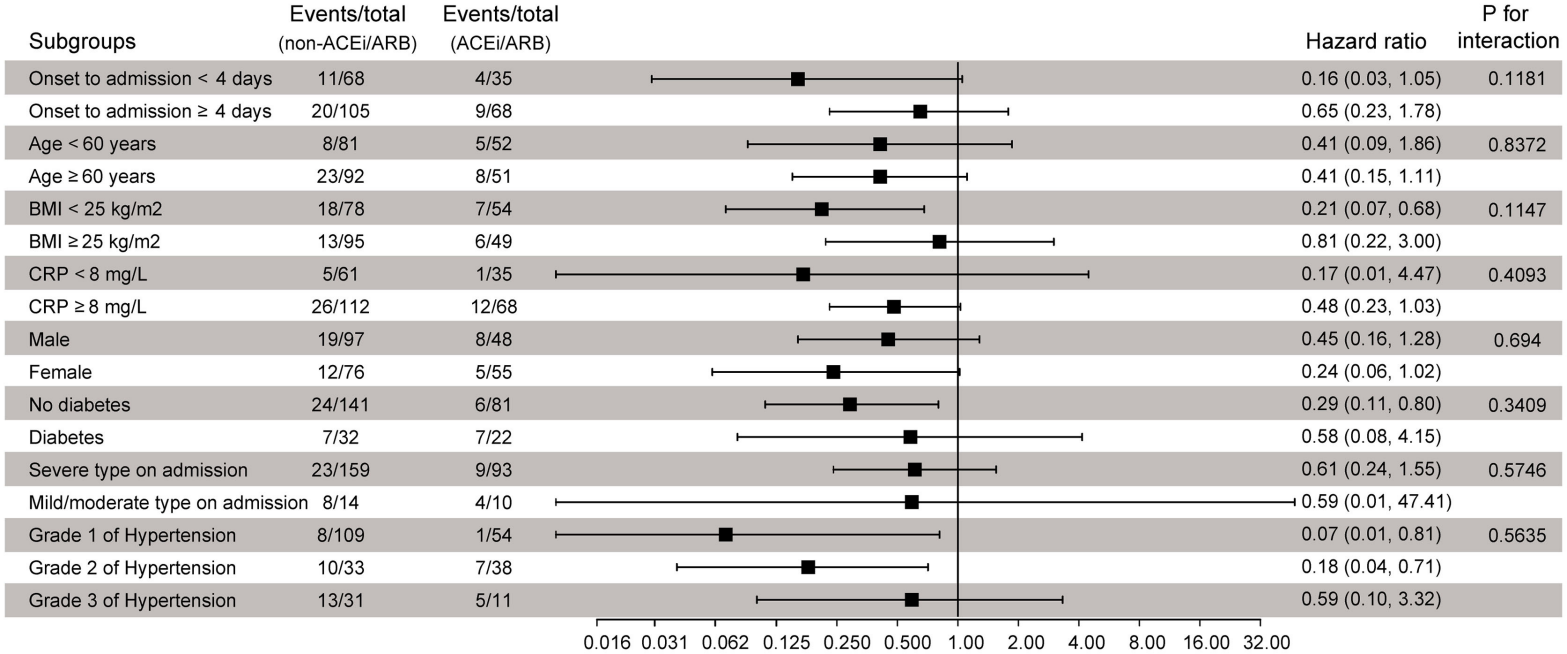
Subgroup analysis of component endpoints according to ACEi/ARB treatment. ACEi, angiotensin-converting enzyme inhibitor; ARB, angiotensin receptor blocker; BMI, body mass index; CRP, C-reactive protein; HR, hazard ratio.

To further confirm whether the observed findings were robust to potential confounders, we performed stratified analyses by prespecified subgroups; all analyses were adjusted for all variables as the Cox regression model except for the stratification variable itself. Compared with the non-ACEi/ARB group, the risk of composite endpoint events probability did not increase in the ACEi/ARB group, with HRs ranging from 0.07 to 0.80 (Figure 3), and no statistically significant interaction was found. In addition, adding the 10 patients who were not taking any antihypertensive drugs in the control group did not change the result; the weighted HR was 0.48 (95% CI, 0.30–0.77; *p* = 0.0022; Supplementary Figure 2). The results of the sensitivity analyses support our main findings.

## Discussion

In this multicenter retrospective study, our results suggest that chronic treatment with ACEi/ARB is not associated with an increased severity of clinical outcome in COVID-19 patients with hypertension. The values of HRs were below 1 in all subgroups considered and after careful adjustments, including an IPTW analysis. In addition, the median progression event time of the ACEi/ARB group was significantly longer than that of the non-ACEi/ARB group (12 vs. 9 days, *p* < 0.001). This finding supported the continued use of RAS inhibitors in COVID-19 patients with hypertension, which provides clinical evidence for the recommendations of international societies.

RAS plays an important role in the pathogenesis and development of hypertension. ACEis and ARBs are commonly used in hypertensive patients as two targeted RAS system inhibitors. There is evidence demonstrating that activation of RAS is associated with acute lung injury in the SARS-CoV-infected model with downregulated ACE2 expression in the lungs, but the lung failure in this setting could be attenuated by treatment with ACEi/ARB (21–23). Furthermore, a systematic meta-study showed that ACEi/ARB can reduce the incidence of community-acquired pneumonia and pneumonia-related mortality (24). A recent study found that angiotensin II was significantly elevated in COVID-19 patients and was in a positive linear correlation with viral load and lung injury (25). Another study also supported the use of ACEi/ARB in improving clinical outcomes of COVID-19 patients with hypertension, as they found that using ACEi/ARB could significantly reduce the level of interleukin 6 while increasing the level of peripheral blood T cells (26).

To assess the potential effects of ACEi/ARB use on these in-hospital patients with COVID-19, we limited our analysis to a cohort of patients with coexisting hypertension and excluded those without hypertension. Several previous studies included the patients without hypertension in the non-ACEi/ARB group and concluded that the use of ACEi/ARB was not related to the severity of the disease (27–30), which may underestimate the effect of ACEi/ARB on patients with COVID-19, since hypertension itself was a risk factor for disease progression (31).

To the best of our knowledge, several observational studies have evaluated the impact of ACEi/ARB use on clinical outcomes in patients with COVID-19 (1, 6–11) and have offered different perspectives. Observational studies may be prone to bias and cannot provide robust results because interventions are not randomly assigned. Despite such shortcomings, observational data represent current clinical practice and apply modern methods to minimize selection bias to assess the effectiveness of clinical interventions and may help guide clinical decision-making. Two recent systematic reviews and meta-analyses concluded that the use of ACEi/ARB is significantly associated with decreased mortality in COVID-19 patients with hypertension but not associated with disease severity (13, 32). These systematic reviews recognized similar limitations, such as research heterogeneity, all studies included were observational, and most studies only adjusted for age and gender without considering other potential confounders and selection bias. Therefore, it is impossible to determine whether ACEi/ARB use is actually effective in SARS-COV-2-infected patients.

Feng et al. (33) first reported from Wuhan that there was a significant difference in ACEi/ARB usage among patients of different severities; the number of severe or critical patients was significantly lower in the ACEi/ARB group than in the non-ACEi/ARB group, but this research did not consider confounding factors, as other studies have done (8, 10). Another multicenter retrospective study performed an analysis among 1,128 COVID-19 patients with preexisting hypertension, which included 188 patients on treatment with ACEi/ARB (7). The effect of ACEi/ARB treatment was analyzed using a multivariate adjustment for confounding variables and propensity score (PS) matching. And results stated that ACEi/ARB was associated with a lower rate of severe outcomes with SARS-CoV-2 infection. These data are in concordance with our results, but the mortality rate in our patients was substantially lower. This discrepancy might result from several factors, i.e., delayed hospitalization after symptom onset in Hubei may lead to disease progression (34). In addition, in every five death cases of COVID-19, only one received invasive mechanical ventilation or further active respiratory support, suggesting that ventilation equipment was limited and intubation was delayed for many patients (35). But the authors did not provide many details about the duration between the onset of symptoms to admission (36) and the grade of hypertension like another study from Korea (9), which were found to be significantly associated with the severity of COVID-19 in our study (Supplementary Table 3). After controlling important confounding factors through multivariate adjustment and IPTW analysis, the results suggested a favorable association of using ACEi/ARB and less severity in COVID-19 patients. Furthermore, sensitivity analyses supported our main finding.

The main advantage of this study is exploring the association between chronic treatment with ACEi/ARB and COVID-19 progression after adjusting the major confounding factors such as the interval between symptom onset to admission and grade of hypertension, and our sample size is relatively larger compared with studies conducted in non-endemic areas in China (14, 15). However, we recognize some limitations. First, due to the relatively lower mortality rate, we could not assess the association between ACEi/ARB use and mortality in COVID-19 patients with hypertension. Since the study was conducted in a non-epidemic pandemic area and there were sufficient medical resources to support the treatment of patients with COVID-19, this study can better reduce confounding factors caused by a shortage of medical resources. Second, the sample size in this study is not big enough. This study included 103 patients receiving ACEi/ARB therapy; only 12 of whom received ACEi. Therefore, subgroup analysis of the differences between the two drugs could not be performed. Third, since patients were not randomly allocated to ACEi/ARB therapy or other regimens, the results may be affected by selection/collider bias. IPTW analysis was used to minimize selection bias, which is a powerful and flexible approach to adjust for collider bias and reduce observational bias and is the best evidence available in observational studies. But IPTW analysis may also have limitations, as this approach may not reflect possible biases in observational studies, and some residual confounding may persist. Fourth, our results were obtained from patients with COVID-19 in non-endemic areas of China. Due to policy reasons, the impact of using ACEi/ARB in other countries/regions on SARS-CoV-2-infected patients needs further study. Whether the current results are applicable to other global populations, long-term prospective studies and randomized clinical trials are still needed to investigate the effects of these treatments.

## Conclusion

In a group of hospitalized COVID-19 patients with preexisting hypertension, chronic treatment with ACEi/ARB does not seem to increase the risk of disease severity after adequate adjustment by IPTW. ACEi/ARB could be continued as antihypertensive therapy for COVID-19 patients with hypertension according to the recommendations of international societies.

## Data Availability Statement

The raw data supporting the conclusions of this article will be made available by the authors, without undue reservation.

## Ethics Statement

This study was approved by the First Affiliated Hospital, College of Medicine, Zhejiang University. Written informed consent for participation was not required for this study in accordance with the national legislation and the institutional requirements.

## Author Contributions

HC and LL: concept and study design. JYu, XShi, JM, FL, JW, XShe, QP, and JYa: data acquisition. JYu and XShi: data analyses. JYu and JM: statistics. JYu: manuscript preparation. JYu, XShi, JM, FL, JW, QP, JYa, HC, and LL: review of the manuscript. All authors approved the final version of the manuscript.

## Conflict of Interest

The authors declare that the research was conducted in the absence of any commercial or financial relationships that could be construed as a potential conflict of interest.
