# Supplementary material for: Association of ACEi/ARB Use and Clinical Outcomes of COVID-19 Patients With Hypertension
Source: Front Cardiovasc Med. 2021 May 31;8:577398. doi: 10.3389/fcvm.2021.577398 (PMC8202940; doi:10.3389/fcvm.2021.577398)

Table S1 Laboratory examination on admission of ACEI/ARB and non-ACEI/ARB groups

|  | Non-ACEI/ARB (n=173) | ACEI/ARB  (n=103) | *P*-value |
| --- | --- | --- | --- |
| Leucocytes (10^9^/L) | 5.40 (4.40-6.50) | 5.19 (3.78-6.47) | 0.265 |
| Neutrophils (10^9^/L) | 3.43 (2.50-4.41) | 3.32 (2.42-4.53) | 0.491 |
| Lymphocytes (10^9^/L) | 1.10 (0.80-1.40) | 1.04 (0.79-1.43) | 0.785 |
| Platelets | 181.00 (139.00-232.50) | 175.00 (144.50-227.00) | 0.974 |
| International normalized ratio | 1.03 (0.99-1.08) | 1.04 (0.98-1.14) | 0.411 |
| Albumin (g/L) | 39.20 (36.00-42.10) | 38.10 (35.15-41.80) | 0.14 |
| Aminotransferase (U/L) | 26.00 (17.00-40.00) | 26.00 (17.50-42.50) | 0.438 |
| Aminotransferase (U/L) | 25.00 (20.00-36.00) | 27.00 (20.00-38.00) | 0.7 |
| Total bilirubin (umol/L) | 11.00 (8.30-14.72) | 10.70 (7.68-15.10) | 0.473 |
| Serum potassium (mmol/L) | 3.90 (3.59-4.28) | 3.87 (3.56-4.20) | 0.614 |
| Serum sodium (mmol/L) | 137.80 (135.00-139.97) | 137.75 (135.44-139.78) | 0.402 |
| Serum potassium (mmol/L) | 4.61 (3.80-5.87) | 4.60 (3.55-6.26) | 0.113 |
| Serum creatinine (umol/L) | 73.00 (60.00-85.00) | 66.40 (53.75-82.85) | 0.884 |
| Creatine kinase (U/L) | 73.00 (51.00-126.00) | 70.00 (51.00-119.50) | 0.79 |
| Lactate dehydrogenase (U/L) | 227.50 (174.00-293.25) | 234.50 (180.25-314.00) | 0.268 |

Abbreviations: ACEI, angiotensin converting enzyme inhibitor; ARB, angiotensin II receptor blocker; SD, Standardized difference.

Table S2. Variables included in the propensity score model

| Variable | Type |
| --- | --- |
| Grade of hypertension | Trinary ( grade 1, grade 2, grade 3) |
| Smoking status | Binary (Yes/No) |
| C-reactive protein | Continuous (mg/L) |
| Body mass index | Continuous (kg/m^2^) |
| Treatment by interferon | Binary (Yes/No) |
| Cardiovascular diseases | Binary (Yes/No) |
| Treatment by arbidol | Binary (Yes/No) |
| Duration from onset to admission | Continuous (days) |
| Chronic liver disease | Binary (Yes/No) |
| Diabetes | Binary (Yes/No) |
| Treatment by Lopinavir/Ritonavir | Binary (Yes/No) |
| Clinical Type on admission | Binary (mild, moderate/severe) |
| Chronic renal disease | Binary (Yes/No) |
| Gender | Binary (Male/Female) |
| Age | Continuous (year) |

Table S3. Hazard ratios (95% CIs) for the composite endpoint for all variables included as covariates in the Cox multivariable model with inverse probability weighting by the propensity score (n=276)

| Variable | **Hazard ratio (95% confidence interval)** | ***P* value** |
| --- | --- | --- |
| Gender (Male vs. Female) | 1.56(0.74, 3.32) | 0.2452 |
| Treatment by ACEI/ARB (Yes vs, No) | 0.41(0.19, 0.88) | 0.0218 |
| Age(years) | 1.02(0.99, 1.06) | 0.213 |
| BMI (kg/m2) | 0.98(0.85, 1.12) | 0.7375 |
| Smoking status (Yes vs, No) | 0.87(0.34, 2.24) | 0.7762 |
| Cardiovascular diseases (Yes vs, No) | 1.39(0.52, 3.71) | 0.5129 |
| Diabetes (Yes vs, No) | 1.27(0.60, 2.70) | 0.533 |
| Chronic liver disease (Yes vs, No) | 2.54(1.01, 6.41) | 0.0484 |
| Chronic renal disease (Yes vs, No) | 2.71(0.51, 14.42) | 0.2438 |
| Treatment by interferon (Yes vs, No) | 0.26(0.02, 0.54) | 0.0003 |
| Treatment by arbidol (Yes vs, No) | 1.09(0.51, 2.32) | 0.8319 |
| Treatment by Lopinavir/Ritonavir (Yes vs, No) | 3.38(1.43, 7.97) | 0.0055 |
| Duration from onset to admission (days) | 0.89(0.82, 0.97) | 0.0082 |
| Clinical Type on admission (mild, moderate vs. severe type) | 4.52(2.01, 10.17) | 0.0003 |
| C-reactive protein (mg/L) | 1.02(1.01, 1.03) | <0.0001 |
| Grade 1 of hypertension | Reference |  |
| Grade 2 of hypertension | 5.73(2.25, 14.59) | 0.0003 |
| Grade 3 of hypertension | 10.97(3.92, 30.69) | <0.0001 |

**Figure S1** Standardised differences of variables included in the propensity score model (n=276).


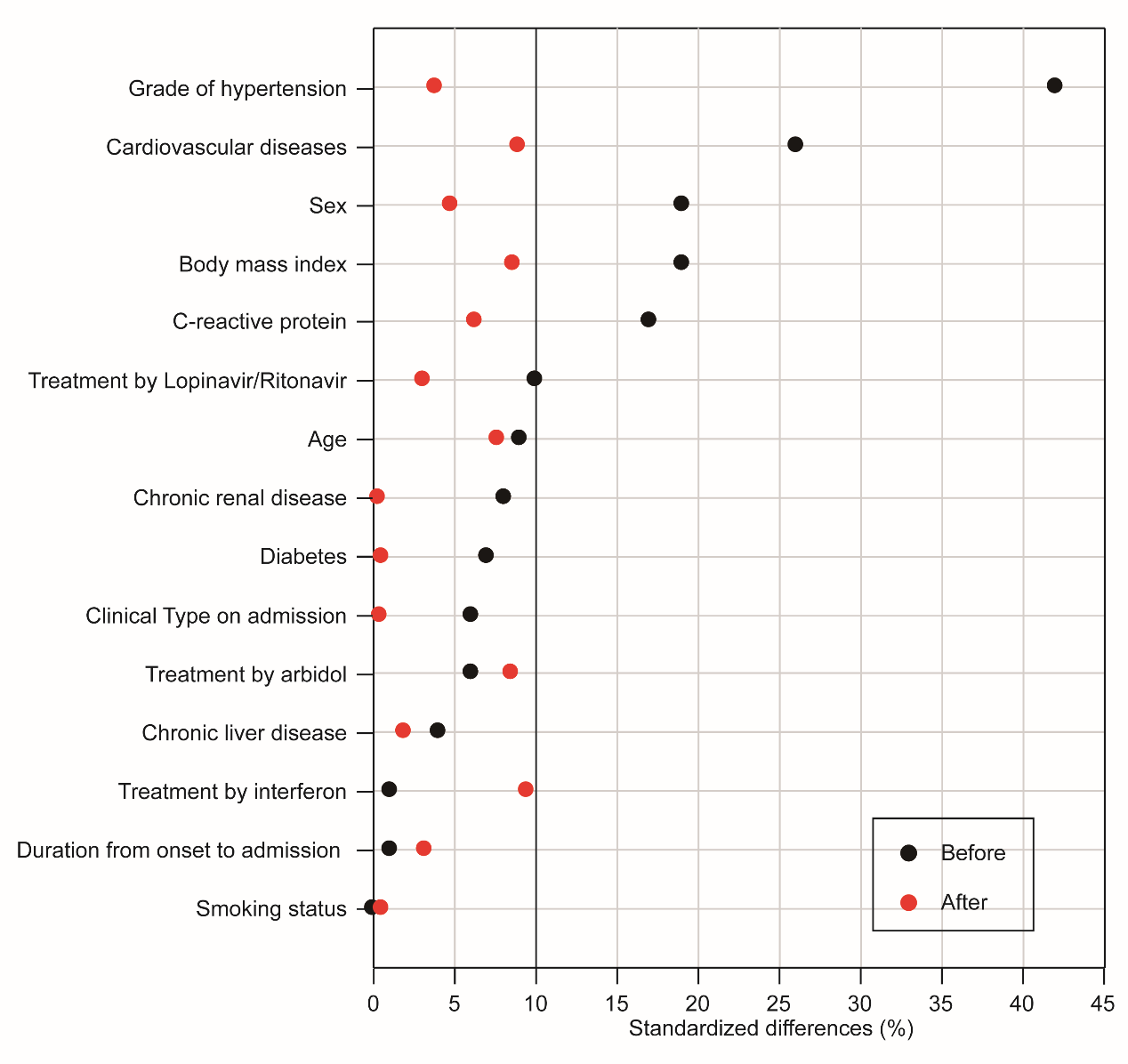


**Figure S2.** The Kaplan-Meier curve for the composite endpoints before (left panel) and after (right panel) IPTW


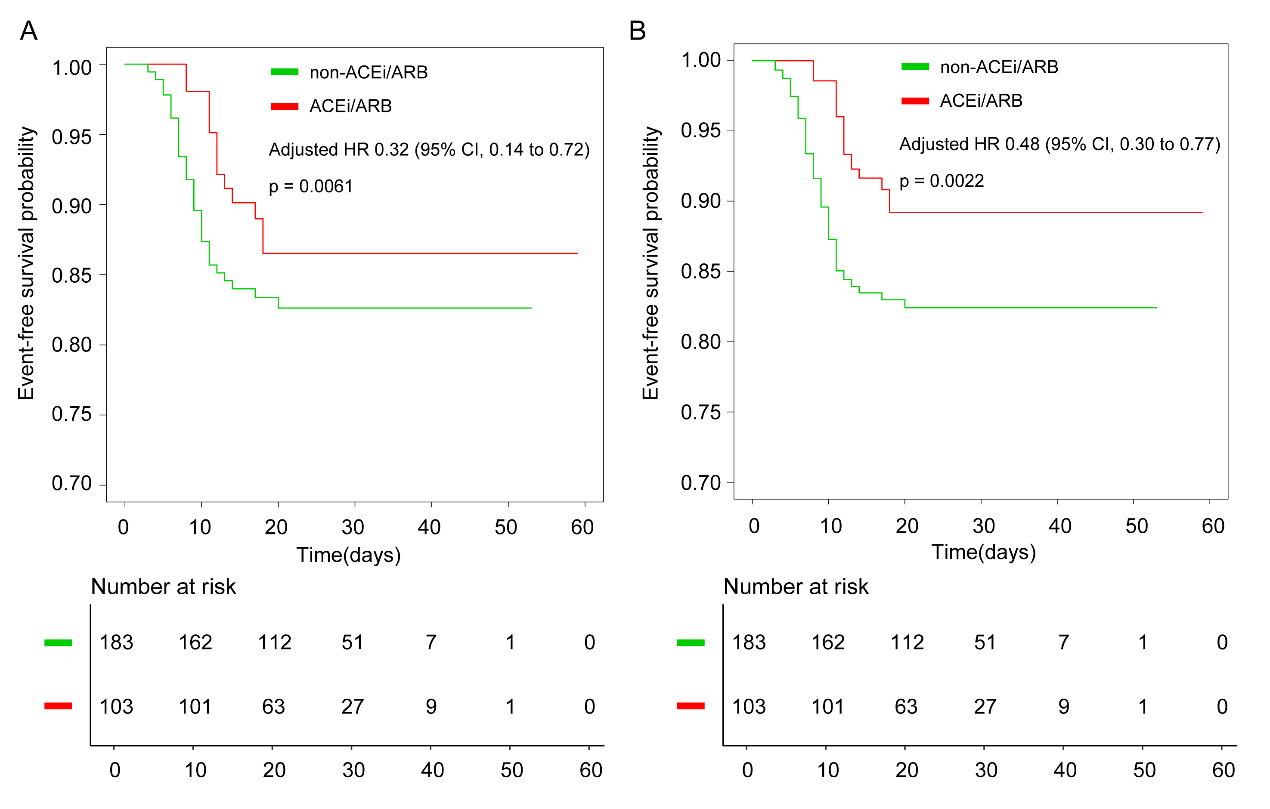

Supplement: Supplementary file 1 [file Data_Sheet_1.docx]
